# The relationship between muscle sympathetic nerve activity and systemic hemodynamics is altered in women with uterine fibroids

**DOI:** 10.14814/phy2.15445

**Published:** 2022-09-19

**Authors:** Ronée E. Harvey, Shannon K. Laughlin‐Tommaso, Elizabeth A. Stewart, Jacqueline K. Limberg, Timothy B. Curry, Michael J. Joyner, Jill N. Barnes

**Affiliations:** ^1^ Mayo Clinic College of Medicine and Science Mayo Clinic Rochester Minnesota USA; ^2^ Department of Anesthesiology Mayo Clinic Rochester Minnesota USA; ^3^ Department of Obstetrics‐Gynecology Mayo Clinic Rochester Minnesota USA; ^4^ Department of Nutrition and Exercise Physiology University of Missouri Columbia Missouri USA; ^5^ Department of Kinesiology University of Wisconsin‐Madison Madison Wisconsin USA

**Keywords:** autonomic, blood pressure, hypertension, uterine leiomyoma

## Abstract

Women with uterine fibroids (UF), benign tumors of the myometrium, have a higher prevalence of hypertension than women without UF. The cause for this relationship is unclear. Muscle sympathetic nerve activity (MSNA) is a regulator of arterial blood pressure, and it is possible that variations in MSNA predispose women with UF to develop hypertension. The purpose of this study was to assess baseline blood pressure and MSNA and the relationships between MSNA and systemic hemodynamics in women with and without UF. We measured blood pressure (brachial intra‐arterial line), MSNA (microneurography), and systemic hemodynamics (total peripheral resistance and cardiac output) at rest in 14 healthy, normotensive, premenopausal women with UF (42 ± 2 years old) and 9 healthy, normotensive, premenopausal women without UF (41 ± 2 years old). Baseline blood pressure and MSNA did not differ between groups (*p* > 0.05 for both). In women with UF, there was a positive correlation between MSNA and total peripheral resistance (*r* = 0.75, *p* = 0.02), as well as a negative correlation between MSNA and cardiac output (*r* = −0.73, *p* = 0.03). In contrast, these relationships were not seen in women without UF (*p* > 0.05 for both relationships). These data suggest that autonomic interactions with systemic hemodynamics, and thus blood pressure regulation, are different in healthy women with UF compared to healthy women without UF.

## Graphical abstract

Muscle sympathetic nerve activity is positively correlated with total peripheral resistance in women with uterine fibroids but not in women without uterine fibroids. Autonomic interactions with systemic hemodynamics, and thus blood pressure regulation, are different in women with uterine fibroids (who are otherwise healthy) compared to healthy women without uterine fibroids.

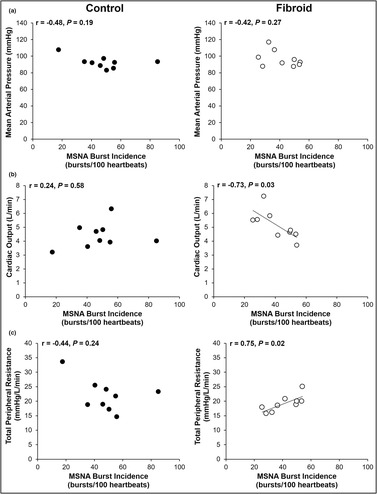

## 1 INTRODUCTION

Hypertension affects approximately one third of people in the United States, and it is necessary to identify those at risk for developing the condition in order to reduce morbidity and mortality of the disease. (Whelton et al., 2018) Uterine fibroids (UF) are benign tumors of the myometrium that affect up to 70% of women by the time of menopause. (Flake et al., 2003; Sparic et al., 2016) Thirty percent of these women experience symptoms of menorrhagia, pelvic pain, and miscarriage. Conditions associated with UF include obesity, diabetes, hypertension, and hypertensive pregnancy disorders. (Sparic et al., 2016; Tak et al., 2016) The prevalence of hypertension in women with UF has been reported to be up to 42%. (Boynton‐Jarrett et al., 2005; Faerstein et al., 2001; Radin et al., 2012; Sivri et al., 2012) UF have been shown to be associated with subclinical atherosclerosis and endothelial dysfunction; (Aksoy et al., 2014; He et al., 2013; Wallace et al., 2014) however, the mechanisms underlying the relationship between UF and hypertension are poorly understood.

The autonomic nervous system provides short‐ and long‐term regulation of blood pressure; and elevated muscle sympathetic nerve activity (MSNA) is observed in individuals with hypertension, sleep apnea, and heart failure. (Charkoudian & Rabbitts, 2009) MSNA and arterial blood pressure are unrelated in young men and women; however, they are positively correlated in older men and women. (Hart et al., 2012) These findings suggest the existence of compensatory mechanisms in young populations that are capable of balancing the influence of MSNA on arterial pressure that are subsequently lost with aging. Because women with uterine fibroids have an increased risk of hypertension, it is possible that this population may show high levels of MSNA and a greater than expected relationship between MSNA and blood pressure. Using a cross‐sectional study design, we sought to determine if the presence of UF was associated with altered autonomic function in normotensive premenopausal women currently diagnosed with UF. We hypothesized that women with UF would have greater baseline blood pressure and MSNA, as well as an altered relationship between MNSA and systemic hemodynamics in comparison to women without UF.

## 2 MATERIALS AND METHODS

### 2.1 Study participants

This study was approved by the Mayo Clinic Institutional Review Board (protocol 13–000596), and the study was performed in accordance with the Declaration of Helsinki. All study participants provided written informed consent prior to participation. Women with a current diagnosis of UF (*n* = 14, 42 ± 2 years old) and women without UF (*n* = 9, 41 ± 2 years old) completed the study. Presence or absence of UF was confirmed by transvaginal ultrasound or magnetic resonance imaging ≤12 months prior to study participation. Individuals were normotensive (brachial cuff blood pressure <130/80 mmHg), (Whelton et al., 2018) nondiabetic, nonobese, nonsmokers, and free of cardiovascular and chronic disease. Women were not taking any medications with the exception of an iron supplement in one woman in the UF group and oral contraceptives. Women were studied during the early follicular phase of the menstrual cycle or placebo phase of oral contraceptive use (*n* = 2 in UF group). Pregnant and breastfeeding women were excluded from the study; pregnancy status was confirmed by a urine pregnancy test within 48 h prior to the study day.

### 2.2 Experimental protocol

Participants had refrained from alcohol, caffeine, and exercise for 24 h prior to the study. Following an overnight fast, women were admitted to the Clinical Research and Trials Unit at the Mayo Clinic, where the ambient temperature was controlled to be 22–24°C. Participants rested in the supine position for 10 min prior to initiation of measurements and throughout the study. A 20‐gauge, 5‐cm catheter, connected to a pressure transducer, was inserted into the brachial artery to measure continuous beat‐to‐beat blood pressure. A 3‐lead ECG was used to continuously record heart rate (Cardiocap/5; Datex‐Ohmeda). One arterial blood sample was collected via a brachial arterial line port at the start of the study for the measurement of female sex hormones.

### 2.3 Measurements

Multiunit MSNA was recorded at the right peroneal nerve posterior to the fibular head using a tungsten microelectrode. A reference electrode was placed subcutaneously ~3 cm from the recording site. The recorded signal was amplified 80,000‐fold, band‐pass filtered (700–2000 Hz), rectified, and integrated (resistance–capacitance integrator circuit, time constant 0.1 s) by a nerve traffic analyzer (662C‐4 Nerve Traffic Analysis System, University of Iowa). A quality recording was identified when taps on the muscle belly or passive muscle stretch evoked mechanoreceptive impulses and when no afferent neural responses were evoked by skin stimuli. (Sundlof & Wallin, 1977) Baseline data were collected across 5‐min. (Briant et al., 2016; Hart et al., 2014)

### 2.4 Data analysis

Data were collected at 250 Hz and stored via an offline computer using the WinDaq software system (DATAQ Instruments). Data were analyzed using the WinCPRS software program (Absolute Aliens). Stroke volume (SV, in ml) was obtained from the brachial blood pressure waveform. Cardiac output (CO, L/min) was calculated as SV × heart rate/1000. Total peripheral resistance (TPR, mmHg/L/min) was calculated as mean arterial pressure/CO. Sympathetic nerve bursts were identified using the automated WinCPRS analysis program. Data were manually corrected by a single investigator (R.E.H.) who was blinded to participant group assignment.

Sympathetic nerve bursts were identified using the automated WinCPRS analysis program that assigns each sympathetic burst to the associated cardiac cycle based on a 1.3‐second burst peak latency from the previous R‐wave. (Carter et al., 2012) A minimum of a 3:1 signal‐to‐noise ratio was used to confirm burst identification. MSNA is expressed as burst frequency (bursts/minute) and burst incidence (bursts/100 heartbeats).

### 2.5 Statistical analysis

All group data are reported as mean ± SEM, unless otherwise noted. Comparative data were analyzed with the Student *t*‐test (SigmaPlot 12.0, Systat Software). Categorical variables (demographic data) were compared using the Fisher Exact test. Pearson correlation analysis was utilized to determine the correlation coefficient (*r*) between MSNA and the hemodynamic variables of mean arterial pressure (MAP), CO, and TPR. A calculated *p*‐value of ≤0.05 was considered significant.

## 3 RESULTS

Participant demographic and baseline data are shown in Table 1. Age, body mass index, and female sex hormone levels did not differ between women with and without UF (*p >* 0.05). Resting hemodynamic values and MSNA levels are shown in Table 2. Baseline brachial cuff and intra‐arterial blood pressures did not differ between groups (*p >* 0.05). SV tended to be greater in women with UF compared to women without UF (*p* = 0.06). CO and TPR did not differ between groups (*p >* 0.05). We were unable to obtain an MSNA recording in 5 women with UF; therefore, MSNA analysis consists of 9 women with UF and 9 controls without UF. Baseline MSNA burst frequency and burst incidence were not different between groups (*p >* 0.05).

**TABLE 1:** Demographic data and characteristics of women with and without uterine fibroids

Variable	Control (*n* = 9)	Uterine fibroid (*n* = 14)	*p* value
Age, years	41 ± 2	42 ± 2	0.38
Body mass index, kg/m^2^	24 ± 1	24 ± 1	0.73
Hormonal contraceptives use, n (%)	0 (0)	2 (14)	0.25
African‐American/Black, n (%)	0 (0)	4 (29)	0.08
Brachial cuff systolic blood pressure, mmHg	107 ± 2	111 ± 2	0.30
Brachial cuff diastolic blood pressure, mmHg	64 ± 2	69 ± 3	0.19
Estradiol, pg/ml	48 ± 8	47 ± 9	0.93
Progesterone, ng/ml	0.54 ± 0.12	0.75 ± 0.16	0.36

*Note:* Values represent mean ± SEM.

**TABLE 2:** Baseline systemic hemodynamics and MSNA levels of women with and without uterine fibroids

Variable	Control (*n* = 9)	Uterine fibroid (*n* = 14)	*p* value
Brachial artery catheter systolic blood pressure, mmHg	127 ± 3	133 ± 4	0.32
Brachial artery catheter diastolic blood pressure, mmHg	75 ± 2	78 ± 2	0.33
Brachial artery catheter pulse pressure, mmHg	52 ± 2	55 ± 2	0.42
Brachial artery catheter mean arterial pressure, mmHg	92 ± 2	96 ± 3	0.31
Heart rate, bpm	59 ± 3	63 ± 2	0.39
Stroke volume, mL	74 ± 3	80 ± 2	0.06
Cardiac output, L/min	4.4 ± 0.3	5.0 ± 0.2	0.10
Total peripheral resistance, mmHg/L/min	22 ± 2	19 ± 1	0.15
MSNA burst frequency, bursts/min	28 ± 4	25 ± 1^a^	0.44
MSNA burst incidence, bursts/100 heartbeats	48 ± 6	41 ± 4^a^	0.34

*Note:* Values represent mean ± SEM. Abbreviation: MSNA, muscle sympathetic nerve activity.

^a^ *n* = 9 in UF group.

**FIGURE 1:**
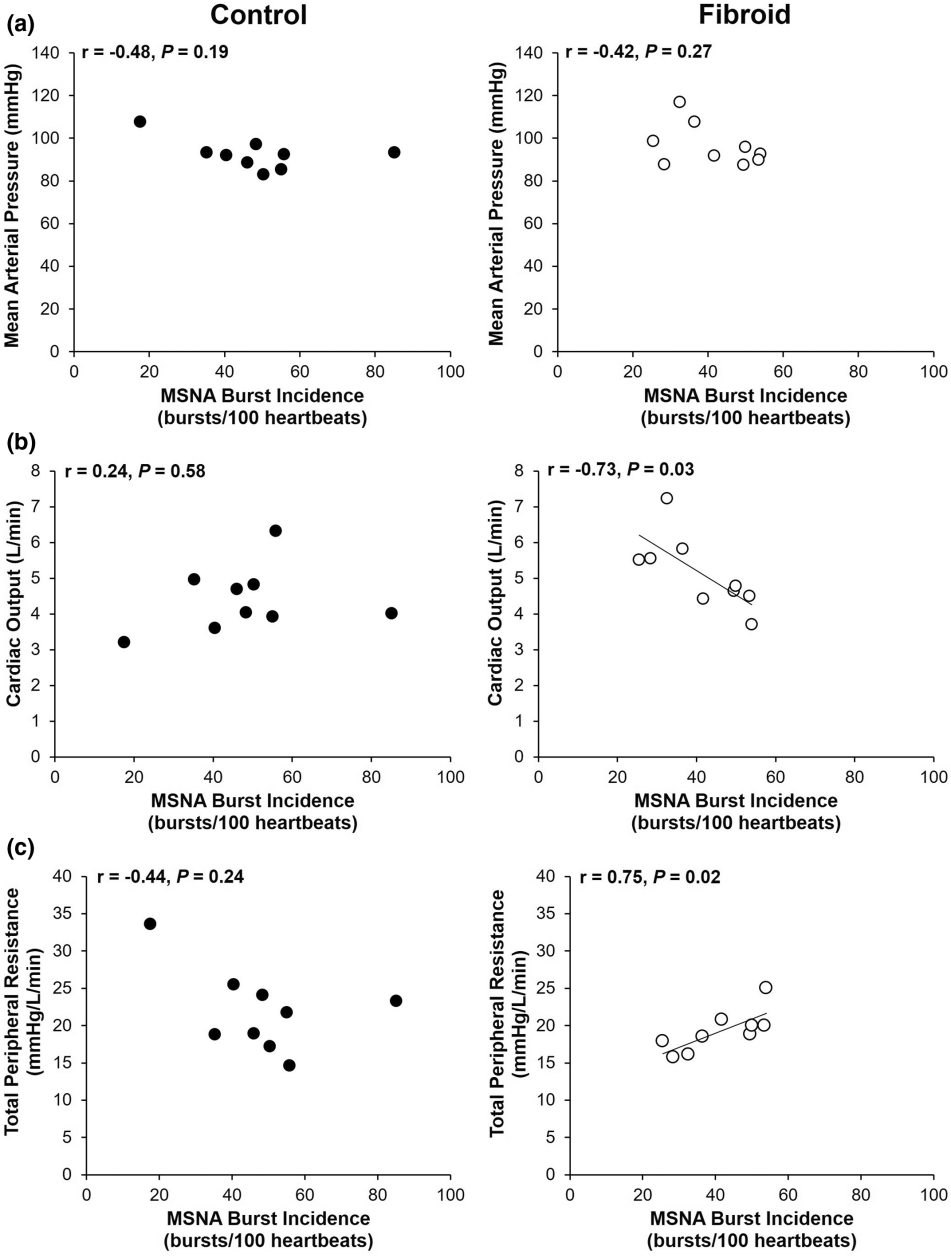
Correlation analyses of the relationships between muscle sympathetic nerve activity (MSNA) burst incidence and mean arterial pressure (a), MSNA burst incidence and cardiac output (b), and MSNA burst incidence and total peripheral resistance (c) in women with (*n* = 9) and without (*n* = 9) uterine fibroids.

Correlation analyses of MSNA burst incidence, MAP, CO, and TPR are shown in Figure 1. There was no significant relationship between MSNA burst incidence and MAP in women with or without UF (*p >* 0.05). A negative relationship between MSNA burst incidence and CO was observed in women with UF (*r* = −0.73, *p* = 0.03); there was no relationship between these variables in women without UF (*r* = 0.24, *p* = 0.58). There was a positive relationship between MSNA burst incidence and TPR in women with UF (*r* = 0.75, *p* = 0.02) while there was no relationship between these variables in women without UF (*r* = −0.44, *p* = 0.24).

## 4 DISCUSSION

To our knowledge, this current study is the first to explore sympathetic nerve activity as a factor in the development of hypertension in normotensive women with UF. Contrary to our hypothesis, there were no differences in baseline blood pressure, MSNA, and systemic hemodynamics between women with and without UF. However, there was a positive relationship between MSNA and TPR and a negative relationship between MSNA and CO in women with UF.

Studies have proposed various models to explain the relationship between UF and hypertension. (Boynton‐Jarrett et al., 2005; Faerstein et al., 2001; Radin et al., 2012; Sivri et al., 2012) There are multiple reports of large‐volume UF causing obstruction of the ureter(s) by mass effect with subsequent hydronephrosis leading to secondary hypertension. (Ascensao et al., 2021; Bansal et al., 2009; Hocutt Jr., 1979; Lai et al., 2009) Removal of such UF by myomectomy was found to a reduce blood pressure in several cases. These studies would suggest that UF precede hypertension development. Data from Wallace et al. suggest that UF can cause hypertension through cellular mechanisms. For example, circulating levels of endothelin‐1, a potent vasoconstrictor, are greater in women with UF than women without UF. Moreover, UF explants express greater levels of endothelin‐1 in comparison to normal adjacent myometrium. (Wallace et al., 2014) Alternatively, hypertension may be the inciting factor for the development of UF. It is proposed that elevated blood pressure damages the smooth muscle cells of the myometrium, leading to tissue proliferation and fibrosis. (Kirschen et al., 2021) Such a response would be similar to that of the vascular smooth muscle in the environment of elevated blood pressure. (Humphrey, 2008)

We suspected that the autonomic system would be the physiological link between UF and blood pressure, as elevated MSNA levels are seen in many individuals with an elevated risk of hypertension (e.g., those with obesity, heart failure, etc.). It has been established that there is no relationship between baseline MSNA and MAP in healthy young women; however, a positive relationship exists between these two variables in older individuals, who are at a higher risk of developing hypertension. (Hart et al., 2012) The present data show no relationship between MSNA and MAP in normotensive women with UF, suggesting that, on this basis alone, their hypertension risk is similar to that of young women without UF.

However, in young men, the lack of relationship between MSNA and MAP is the result of two compensatory relationships: (1) MSNA burst incidence and CO are negatively correlated to each other, and (2) MSNA burst incidence and TPR are positively correlated to each other; (Hart et al., 2011) thus, any sympathetically mediated peripheral vasoconstriction is matched with a reduction in CO. In contrast, neither of these relationships exist in healthy young women, as is further confirmed in the present investigation. These sex‐specific differences are likely due to reduced β‐adrenergic receptor‐mediated vasodilation in young men relative to young women. (Hart et al., 2011; Kneale et al., 2000) Thus, the present finding that women with UF exhibited MSNA‐CO and MSNA‐TPR relationships that are similar to those seen in young men, suggesting lower β‐adrenergic receptor‐mediated vasodilation in this population as well. However, this needs to be studied directly.

Interestingly, cell and tissue composition may provide insight into the relationship amongst UF, blood pressure, and sympathetic nerve activity. Telocytes are interstitial cells found throughout many organs, including the uterus and blood vessels. (Kondo & Kaestner, 2019) These cells are associated with nerve fibers, as well as myometrial cells (myocytes) and the smooth muscle layer of the vasculature. Telocytes modulate cell‐to‐cell signaling, smooth muscle function, angiogenesis, and tissue organization. (Cretoiu & Popescu, 2014; Kondo & Kaestner, 2019) Work by Aleksandrovych et al. examined telocytes and autonomic innervation in UF tissue samples versus normal myometrium. (Aleksandrovych et al., 2019) UF tissue was found to have a lower density of telocytes with a greater density of nerve tissue compared to normal myometrial tissue. The investigators concluded that the abnormal densities of these cell types within UF may contribute to UF pathophysiology. However, in what manner the density of telocytes and nerve fibers relates to that of the peripheral vasculature (and MSNA activity) is not known. Perhaps the concentration of telocytes in UF tissue is not correlated with that of the peripheral vasculature, or MSNA levels, in normotensive women with UF; however, an association might be observed in hypertensive women with UF. Such findings would suggest a role for telocytes in the relationship between UF and hypertension with possible influence by the peripheral vasculature and/or MSNA. Future, long‐term investigations are required to explore these hypotheses.

An alternative explanation for the relationship between UF and hypertension is that a common factor contributes to the pathophysiology of both conditions. For example, there is evidence that disruptions in the renin‐angiotensin‐aldosterone system promote the development of UF and hypertension. Both aldosterone and angiotensin II cause cell proliferation and angiogenesis, and angiotensin II also leads to vasoconstriction. (Armanini et al., 2018) Using an in vitro model, Isobe et al. reported proliferation of rat leiomyoma cells with separate treatments of aldosterone and angiotensin II; however, treatment with a mineralocorticoid receptor antagonist suppressed this activity. (Isobe et al., 2010) In a population of hypertensive women, those who used an angiotensin‐converting enzyme inhibitor (ACE‐i) had a reduced odds of clinically significant UF. (Fischer et al., 2021) This information suggests not only a common pathophysiology for UF and hypertension but also a possible avenue for therapy. We did not observe any difference in serum renin, aldosterone, or ACE levels between the present groups of women (data not shown).

### 4.1 Study limitations

This study is limited by our small sample size. Because we were not able to obtain MSNA recordings in all study participants, the power to detect a difference in MSNA burst frequency between groups was 50% and was 77% for MSNA burst incidence. Additionally, our study was cross‐sectional in nature; thus, we are unable to determine if normotensive women with UF who show a positive TPR‐MSNA relationship will develop hypertension. A longitudinal study is necessary to determine if this altered relationship is predictive of hypertension development in this population.

To limit confounding factors in our study, women with cardiovascular and other systemic co‐morbidities were excluded from participation. Therefore, it may be difficult to generalize our findings to a larger population of women with UF. Recent studies have highlighted the importance of having appropriate representation of populations in research and better understanding the interaction of racial background, socioeconomic status, and disease. (Bunsawat & Robinson, 2021) Despite this limitation, our study did include a number of women identifying as African‐American/Black in the UF group, which is important due to the significant burden of UF in this demographic. (Eltoukhi et al., 2014) Although our sample size was small, the present study has identified a novel physiological relationship which may provide insight into the poorly understood link between UF and hypertension.

Taken together with previous studies, our data further support the inclusion of women with uterine fibroids as a population of interest who is at an increased risk for developing hypertension and cardiovascular disease. However, this is the first study to consider autonomic function in the regulation of blood pressure in women with UF. Our report of differing relationships between MSNA, CO, and TPR in women with UF, suggests that blood pressure regulation may be different in normotensive women with UF in comparison to women without UF.

## AUTHOR CONTRIBUTIONS

R.E.H., S.K.L., E.A.S., M.J.J., and J.N.B. conceived and designed research; R.E.H., J.K.L., T.B.C., M.J.J., and J.N.B. performed the experiments; R.E.H. analyzed the data; R.E.H. and J.N.B. interpreted results of experiments; R.E.H. drafted the manuscript and prepared figures; R.E.H., S.K.L., E.A.S., J.K.L., T.B.C., M.J.J., and J.N.B. edited, revised, and approved the final version of the manuscript.

## FUNDING INFORMATION

This work was supported by American Heart Association predoctoral fellowship 14PRE18040000, National Institutes of Health grants HL83947 and HL118154, and the National Center for Advancing Translational Sciences (NCATS) UL1 TR000135.

## CONFLICT OF INTEREST

None.

## ETHICS STATEMENT

This study was approved by the Mayo Clinic Institutional Review Board. All study participants provided written informed consent prior to participation.
